# Supplementary figures and images for: Reduction of Amyloid Burden by Proliferated Homeostatic Microglia in Toxoplasma gondii-Infected Alzheimer’s Disease Model Mice
Source: Int J Mol Sci. 2021 Mar 9;22(5):2764. doi: 10.3390/ijms22052764 (PMC7975980; doi:10.3390/ijms22052764)

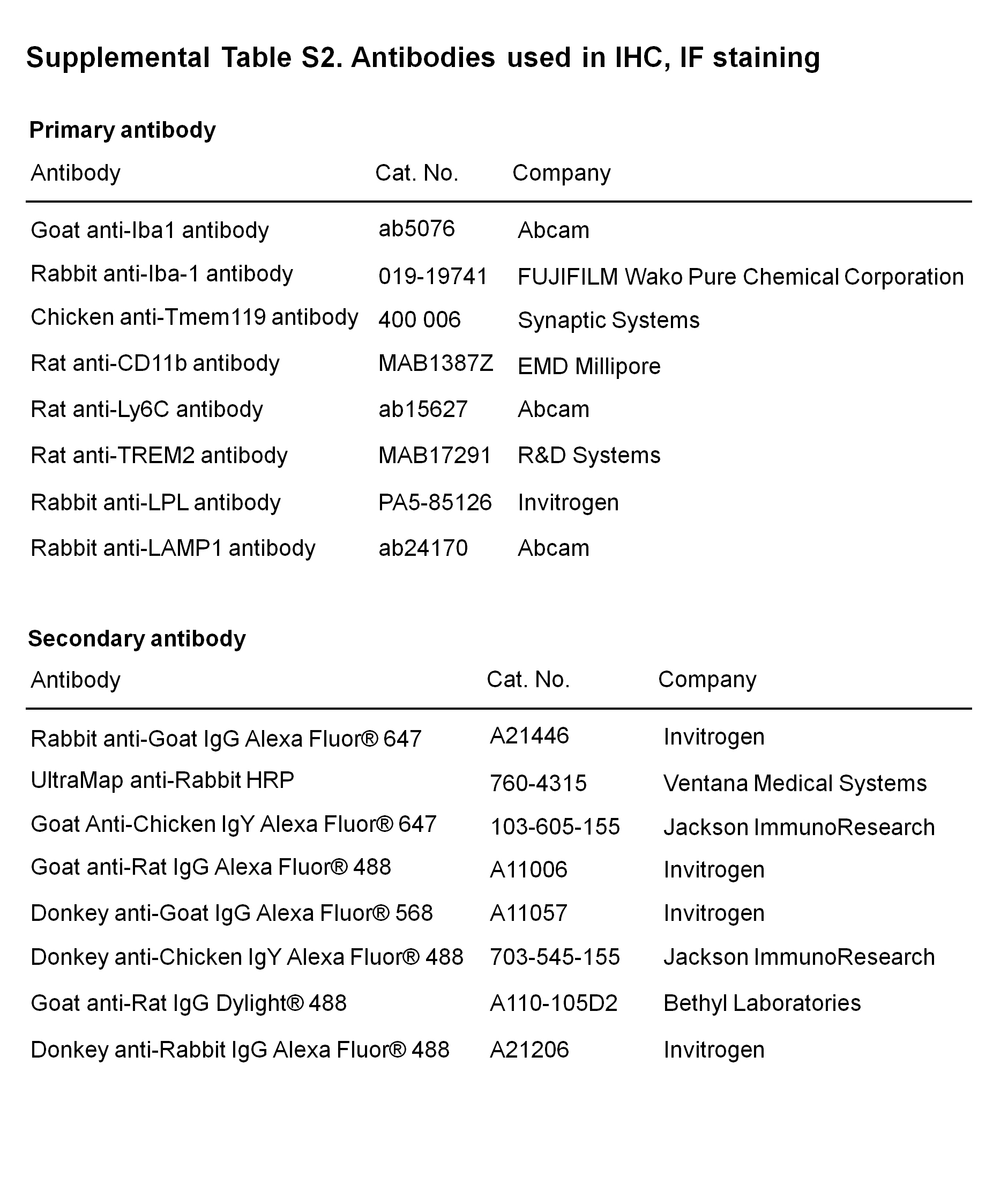

Supplement: Supplementary file 1 [file ijms-22-02764-s001.zip › Supplemental Table S2.jpg]

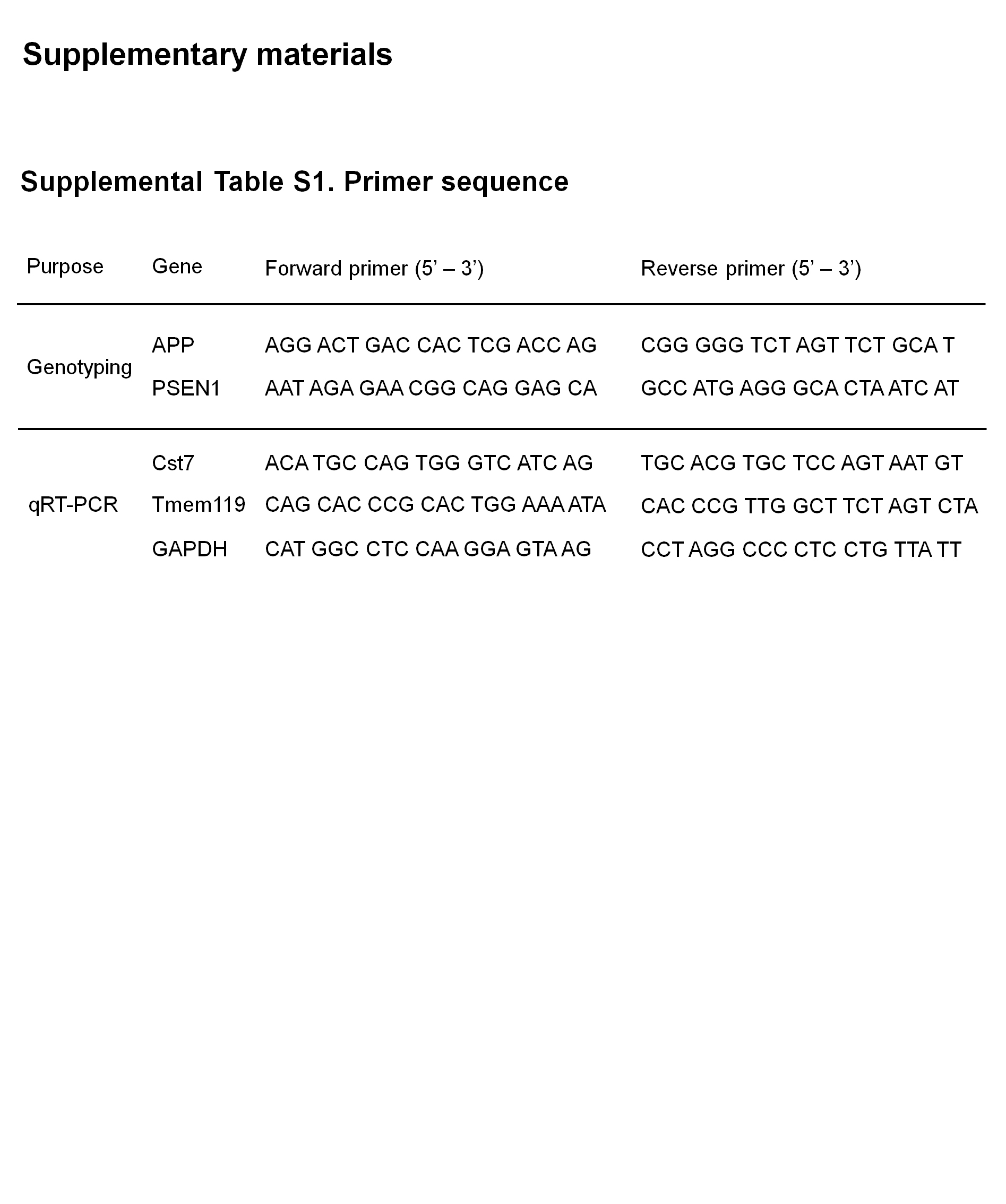

Supplement: Supplementary file 1 [file ijms-22-02764-s001.zip › Supplemental Table S1.jpg]
